# FcMBL magnetic bead-based MALDI-TOF MS rapidly identifies paediatric blood stream infections from positive blood cultures

**DOI:** 10.1371/journal.pone.0276777

**Published:** 2022-11-22

**Authors:** Kerry Anne Kite, Sahil Loomba, Thomas J. Elliott, Francis Yongblah, Shanda L. Lightbown, Thomas J. Doyle, Lily Gates, Dagmar Alber, George A. Downey, Michael T. McCurdy, James A. Hill, Michael Super, Donald E. Ingber, Nigel Klein, Elaine Cloutman-Green

**Affiliations:** 1 Great Ormond Street Institute of Child Health, London, United Kingdom; 2 Department of Mathematics, Imperial College London, London, United Kingdom; 3 Department of Physics and Astronomy, University of Manchester, Manchester, United Kingdom; 4 Department of Mathematics, University of Manchester, Manchester, United Kingdom; 5 Great Ormond Street Hospital, London, United Kingdom; 6 Wyss Institute for Biologically Inspired Engineering, Harvard University, Boston, MA, United States of America; 7 BOA Biomedical Inc., Cambridge, MA, United States of America; 8 Harvard John A. Paulson School of Engineering and Applied Sciences, Harvard University, Cambridge, MA, United States of America; 9 Vascular Biology Program and Department of Surgery, Boston Children’s Hospital and Harvard Medical School, Boston, MA, United States of America; Khoo Teck Puat Hospital, SINGAPORE

## Abstract

Rapid identification of potentially life-threatening blood stream infections (BSI) improves clinical outcomes, yet conventional blood culture (BC) identification methods require ~24–72 hours of liquid culture, plus 24–48 hours to generate single colonies on solid media suitable for identification by mass spectrometry (MS). Newer rapid centrifugation techniques, such as the Bruker MBT-Sepsityper^®^ IVD, replace culturing on solid media and expedite the diagnosis of BCs but frequently demonstrate reduced sensitivity for identifying clinically significant Gram-positive bacterial or fungal infections. This study introduces a protocol that utilises the broad-range binding properties of an engineered version of mannose-binding lectin linked to the Fc portion of immunoglobulin (FcMBL) to capture and enrich pathogens combined with matrix-assisted laser desorption-ionisation time-of-flight (MALDI-TOF) MS for enhanced infection identification in BCs. The FcMBL method identified 94.1% (64 of 68) of clinical BCs processed, with a high sensitivity for both Gram-negative and Gram-positive bacteria (94.7 and 93.2%, respectively). The FcMBL method identified more patient positive BCs than the Sepsityper^®^ (25 of 25 vs 17 of 25), notably with 100% (3/3) sensitivity for clinical candidemia, compared to only 33% (1/3) for the Sepsityper^®^. Additionally, during inoculation experiments, the FcMBL method demonstrated a greater sensitivity, identifying 100% (24/24) of candida to genus level and 9/24 (37.5%) top species level compared to 70.8% (17/24) to genus and 6/24 to species (25%) using the Sepsityper^®^. This study demonstrates that capture and enrichment of samples using magnetic FcMBL-conjugated beads is superior to rapid centrifugation methods for identification of BCs by MALDI-TOF MS. Deploying the FcMBL method therefore offers potential clinical benefits in sensitivity and reduced turnaround times for BC diagnosis compared to the standard Sepsityper^®^ kit, especially for fungal diagnosis.

## Introduction

**Fig 1:**
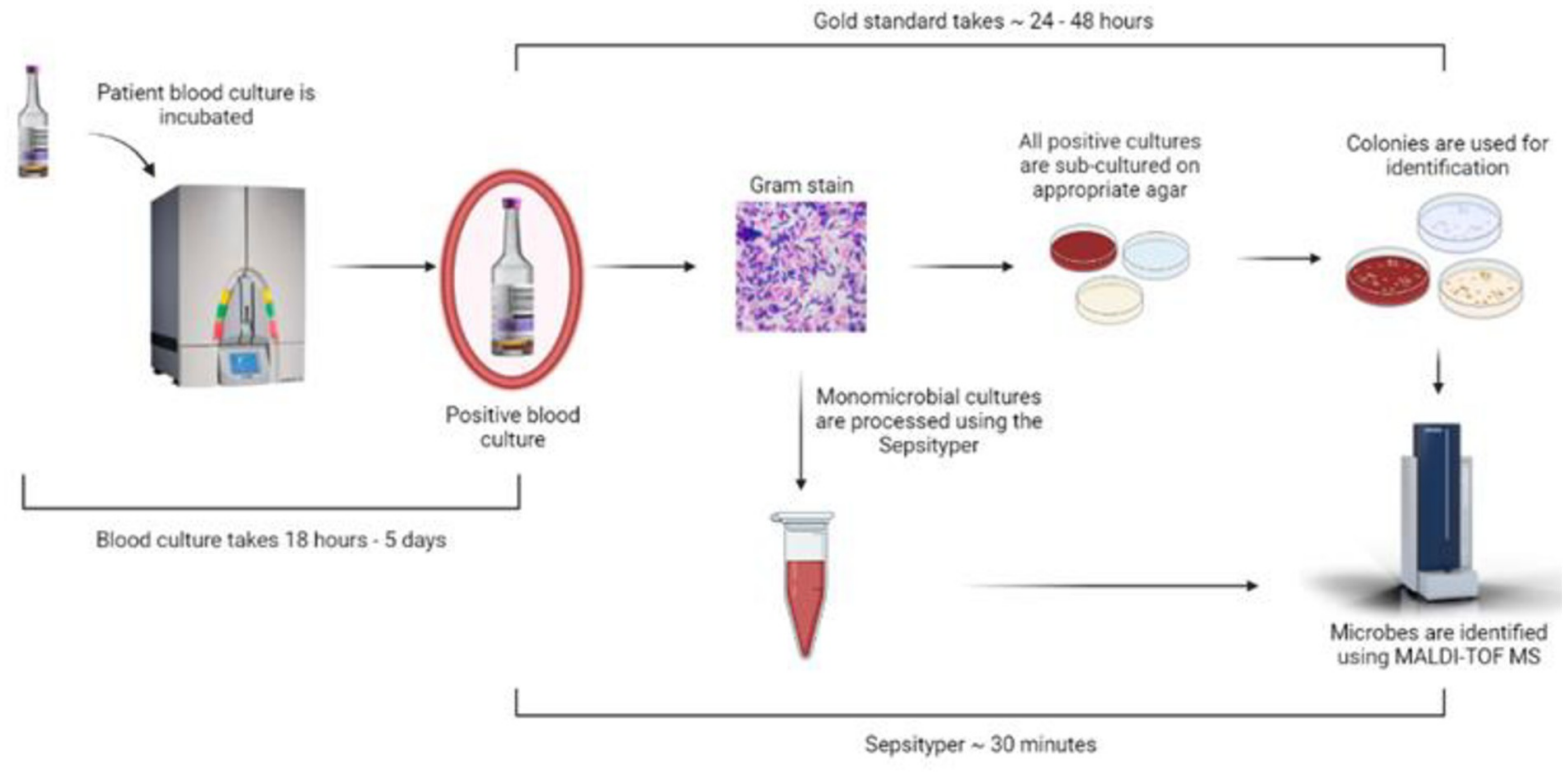
Patient enrolment. Of the 68 patient samples, 25 had Sepsityper analysis performed as part of routine diagnostics.

Due to the wide breadth of pathogens causing BSIs and despite considerable improvements in their diagnosis and treatment, mortality rates remain high, particularly in paediatric settings [1, 2]. Early and accurate pathogen identification is thus essential to optimise patient care. Rapid and adequate antimicrobial therapy is crucial for positive outcomes; this is particularly relevant in septic patients where outcomes directly correlate with the time until the appropriate antimicrobial therapy can be selected [3–5]. Primary amplification and detection of organisms present within blood samples are currently undertaken using blood cultures (BCs). Typically, patient blood is added to paediatric and anaerobic culture bottles and incubated on an automated blood culture instrument for up to five days or until changes are detected in pH or carbon dioxide (CO_2_) associated with microbial growth (Fig 1). Gram strains are then undertaken to provide preliminary phenotypic identification of single or multiple microbes. Further pathogen identification requires solid agar subcultures, further delaying definitive identification by 24–48 hours. But in recent years MALDI-TOF MS has expedited this process, supporting organism identification from single colonies on solid agar within minutes for a relatively small cost [6, 7] (Fig 1). This process requires an average of 24–72 hours for BC growth and a further 24–48 hours for subsequent subculture onto solid agar.

More recent innovations have expanded the use of MALDI-TOF MS to support rapid identification from the positively flagged blood culture bottles using the Bruker Sepsityper^®^ kit (Bruker Daltonik, Bremen, Germany). The Bruker Sepsityper^®^ kit detects Gram-negative bacteria with high sensitivity (83–98%), though detection rates of Gram-positive bacteria and yeast are lower (58–80% and 57–82%, respectively) [6–12]. The Sepsityper^®^ separates microbes from blood using host cell lysis and centrifugation; however, host proteins released during the lysis and centrifugation of blood can introduce noise to MALDI-TOF MS spectra, reducing pathogen identification scores [13].

Mannose-binding lectin (MBL) is an important opsonin of the lectin pathway and a key complement activator, particularly in children [14]. MBL binds various sugar motifs, including mannose, mannan, and *N*-acetylglucosamine, in a calcium-dependent manner [15] and exhibits broad-spectrum capture of Gram-negative and Gram-positive bacteria, bacterial products (e.g., endotoxin), and fungi [14, 15]. FcMBL is a recombinant protein containing the neck and the functional carbohydrate recognition domains of MBL (natural opsonin) fused to the Fc region of human IgG1 [16]. FcMBL was engineered to take advantage of the high expression and easy purification of Fc containing fusion proteins, while also removing coagulation and complement activation of the native MBL protein [16]. FcMBL conjugated to magnetic beads has been shown to bind over 200 different pathogens, including Gram positive and negative bacteria, fungi, viruses, and parasites, as well as toxins and used to successfully isolate pathogens from infected joints and bovine blood [17, 18]. Despite previous studies demonstrating a low affinity of natural MBL for binding to Gram-negative bacteria such as *E*. *coli*, FcMBL has been shown to possess an enhanced binding [19, 20] particularly after pathogen disruption by either mechanical or antibiotic therapy. LC MS MS characterization of FcMBL bead captured material identified 88% of E coli proteins in the captured fraction, while 53% of S aureus proteins were in the FcMBL bead captured fraction [21]. FcMBL conjugated to haemodialysis filters, successfully removed fragmented bacteria and endotoxin to purify septic blood [22].

Utilising this FcMBL magnetic capture capacity, we designed an assay for bacterial and yeast identification directly from positive blood cultures with an aim to decrease time to organism identification and increase assay sensitivity in comparison to other available rapid identification processes. This study compares this new method of using FcMBL-conjugated beads against the Bruker Sepsityper^®^ kit (Catalogue number 1834338) for rapid identification using MALDI-TOF MS within a clinical diagnostic laboratory, showing favourable results.

## Materials and methods

### Chemicals and media

MALDI-TOF MS reagents were purchased from Bruker Daltonics, Coventry, UK. All HPLC-grade chemicals were purchased from Fluka (Honeywell). Agar and BHI media were purchased from Oxoid (Thermofisher, UK). Bactec Plus Aerobic/F (paediatric) and Bactec Lytic/10 Anaerobic/F blood culture bottles were purchased from Becton Dickinson, UK. FcMBL magnetic beads were produced using biotinylated FcMBL and one micron Streptavidin beads (Thermofisher) as previously described [14] and were provided by the Wyss Institute and BOA Biomedical.

### Microbial culture

Bacteria and yeast were cultured in 10mL of BHI medium at 37°C in 5% CO_2_ for 14 hours shaking at 190 rpm. Clinical isolates collected from Great Ormond Street Hospital (GOSH) of *Staphylococcus aureus (1)*, *Escherichia coli (1)*, *Klebsiella pneumoniae (1)*, *Pseudomonas aeruginosa (1)*, *Pseudomonas putida (1)*, and *Enterococcus faecalis (1)* were cultured as above. A total of 24 strains were also collected from the hospital that had been formerly isolated from blood cultures and were implicated in candidemia: *Candida albicans* (6), *Candida guilliermondii* (3), *Candida glabrata* (3) *Candida parapsilosis* (4), *Candida krusei* (1), *Candida kefyr* (3), *Candida auris* (3), and *Candida tropicalis* (1). Colony forming units (CFUs) were calculated by reading absorbance at 600nm and viable counts were performed for exact numbers.

### Experimental design and samples

**Fig 2:**
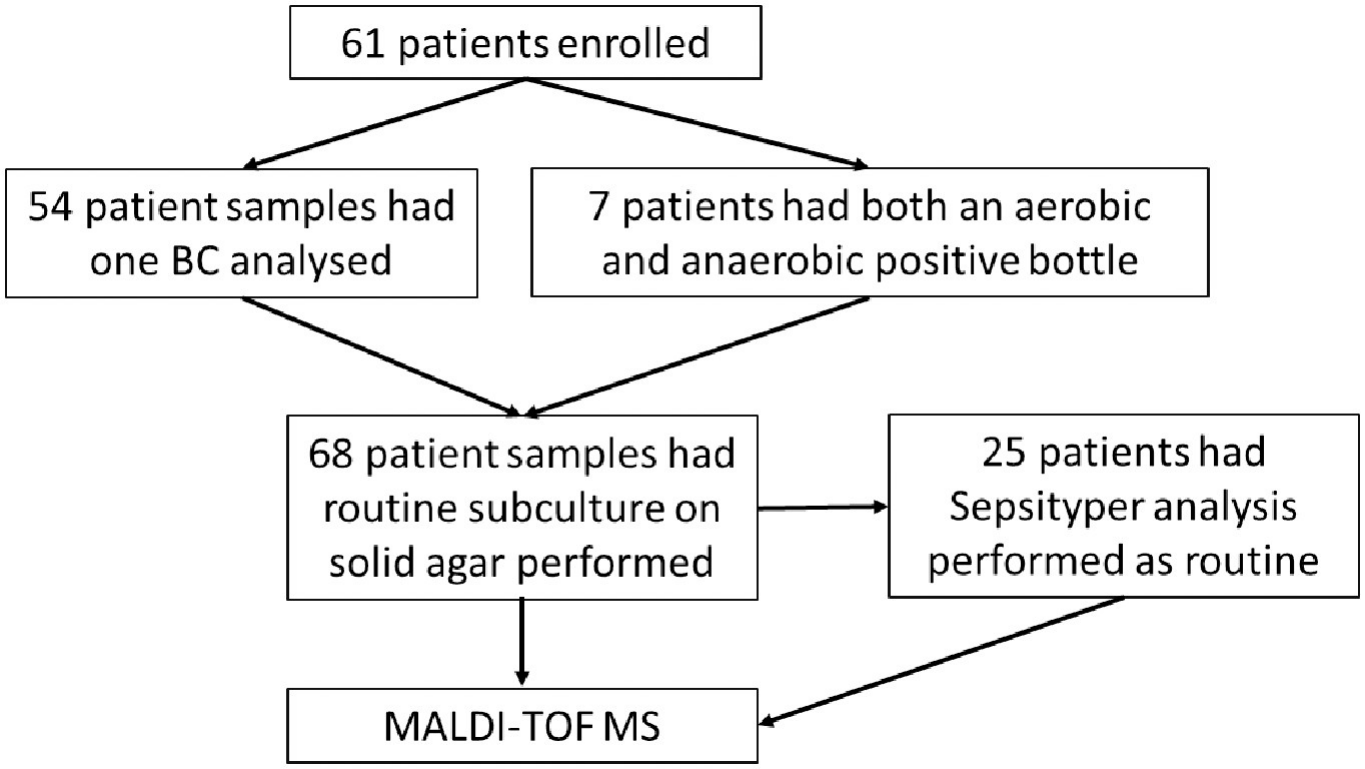
Overview of clinical microbiology SOP.

A blinded clinical evaluation of the FcMBL beads took place at GOSH, London, a major children’s tertiary hospital, between May 2021 and January 2022. Positive BCs detected by the Bactec 9240 automated incubation system (Becton Dickinson) were selected. Inclusion criteria consisted of a positive Gram stain and confirmation of monomicrobial status (Fig 1). Only one positive culture bottle from each patient was selected unless patients had both a positive lytic/10 anaerobic/F and Plus aerobic/F bottle (Fig 2). Clinical samples were processed by clinical staff according to GOSH protocol which for selected samples included the Sepsityper^®^ kit analysis. The samples were also processed blindly with the FcMBL method, and scores and identification were compared after MS analysis. For spiked cultures ~3-10mL of healthy volunteer blood was taken into culture bottles and spiked with ~10CFU and incubated until positive. All investigations were performed in accordance with the Great Ormond Street Hospital’s Research governance policies and procedures. Anonymised residual samples originating from the UK were used in accordance with the Human Tissue Act and the RCPath guidelines for assay development and validation. Under this guidance no consent was required for the use of fully-anonymised residual samples.

### Sepsityper^®^ processing

**Fig 3:**
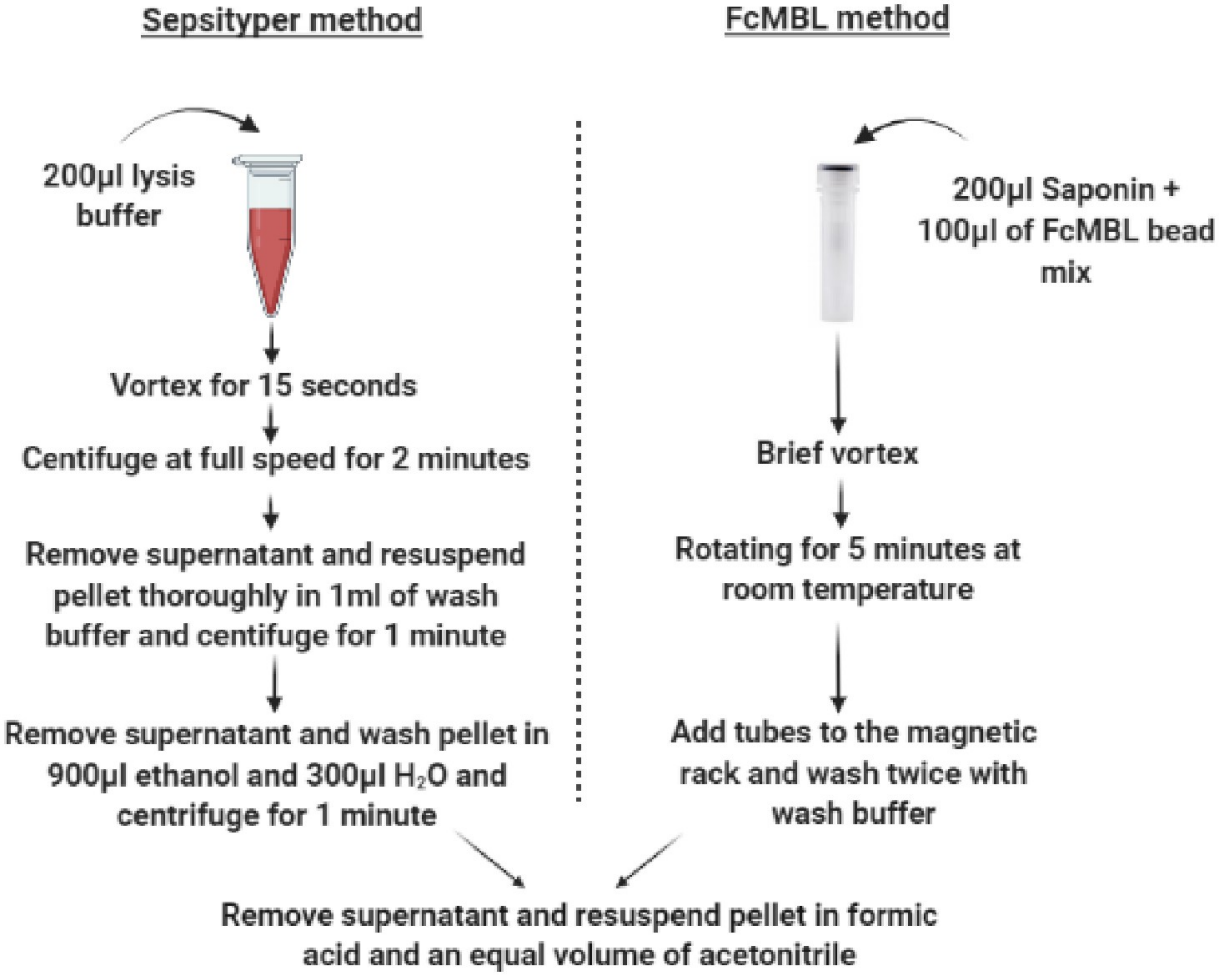
Comparison of the FcMBL and Sepsityper^®^ protocols. Time to identification is ~7 minutes for the full Sepsityper^®^ workflow compared to ~9 minutes for the FcMBL protocol.

One millilitre (mL) of clinical sample was processed according to the full Sepsityper^®^ kit manufacturer instructions by clinical staff at GOSH (Fig 3). Briefly, 200μL of Sepsityper^®^ buffer was added to each sample and vortexed for 10 seconds then centrifuged for two minutes at 12,100*g*. The pellet was washed twice in one mL of Sepsityper^®^ wash buffer and centrifuged for one minute. The supernatant was removed and washed with a mixture of 900μL of 100% ethanol and 300μL of HPLC-grade H_2_O. The sample is then centrifuged 12,100*g* for one minute. After centrifugation, the supernatant was removed, and proteins were extracted with 70% formic acid (FA) and 100% acetonitrile (ACN) (FA/ACN).

### FcMBL processing

One mL of positive spiked donor blood or clinical sample were incubated for five minutes with 100μL of 5% saponin (Millipore) and 100μL (5μg) of FcMBL beads with 10mM glucose and Tris Buffered Saline with Tween (TSBT) 5mM Ca^2+^ at 22°C [20]. The tubes were then placed in a magnetic holder for one minute (New England Biolabs) and the supernatant removed before being washed twice with one mL of HBSS (Life Technologies) (Fig 3). The beads were then eluted into 10μL FA for bacteria and 20μL for yeast and an equal volume of ACN. Identification of fungal or bacterial infection was confirmed using routine Gram staining for clinical samples.

### Direct identification with MALDI-TOF MS of positive blood cultures

Microbial proteins extracted from the Sepsityper^®^ and FcMBL method were layered onto a polished steel plate and left to air dry. Each spot was layered with one μL of alpha-cyano-4-hydroxy cinamic acid (HCCA) matrix (10mg/ml) and left to dry. Plates were analysed within four hours of the matrix being applied. Using the Bruker Biotyper, samples were measured with the default settings for spectra acquisition and processing. The spectra were generated in linear positive ion mode in a mass range of 2-20kDa using a 337nm nitrogen laser with a frequency of 60Hz. The data were analysed automatically with the MALDI Biotyper Realtime classification and Biotyper software version 3.0. Scores of ≥1.70 were considered sufficient for genus level and scores of ≥2.00 indicated species level confidence.

### Magnetic bead enrichment versus centrifugation

*E*. *coli* (NCTC 10418) in sterile PBS was centrifuged and compared with FcMBL-processed *E*. *coli* of the same concentrations spiked into Bactec Plus Aerobic/F blood. Control samples were centrifuged for 10 minutes at 12,100*g* to establish pellets.

### Data analysis

As the clinical samples used for assay optimisation were residual, no patient identities were used, and no further ethical approval was required. MS data were processed and analysed in FlexAnalysis 3.4 software.

Statistical analysis was carried out using GraphPad Prism v9.4.1, to perform McNemar’s test comparing positive/negative identification of pathogens using both methods, and a Wilcoxon matched-pairs signed-rank test on the raw log scores. A significance level of *P*<0.05 was used for all analyses.

Spectral similarities between pairs of spectra were determined using cosine similarity. To account for slight imperfections in the precise location of the observed peaks in the MS we discretised the raw spectra for each sample into *B* bins, with any pair of peaks in a given bin differing by at most Δ. Thus, Δ encodes the assumed level of measurement precision. To ensure stability of the binning a data-driven approach was adopted, wherein all peaks were assigned to bins via agglomerative clustering using the furthest-neighbour criterion [23, 24] with a distance threshold of Δ, ensuring that each bin has width Δ or less. The intensities of peaks assigned to the same bin were then summed to produce a “spectral vector” of dimension *B* for each sample. Finally, the spectral similarity between two samples is given by the cosine similarity between their spectral vectors. The cosine similarity of two vectors *a* and *b* of dimension *B* is given by $\text{S}\left(a,b\right)=\sum_{j=1}^{B}a_{j}b_{j}/\left(\sqrt{\sum_{j=1}^{B}a_{j}^{2}}\sqrt{\sum_{j=1}^{\text{B}}b_{j}^{2}}\right)$.

The spectral similarities between all spiked samples were calculated and visualised via clustered heatmaps (Figs 6 and 7), where sample clusters were inferred via agglomerative clustering based on the spectral similarities using the group-average criterion [24]. Since this method yields a hierarchy of sample clusters, an ‘optimal’ ordering of samples was determined wherein the sum of similarities between adjacent samples is minimised [25], and the rows and columns of the heatmaps were arranged as per the optimal ordering. Consequently, the quality of clusters induced by cosine similarity with respect to species identification can be judged by observing whether samples of the same species appear contiguously in the optimal ordering, i.e., adjacent to each other in the heatmap visualisations.

Because the real precision is unknown, an ‘optimal’ value of Δ was determined by calculating the optimal ordering for a range of values of Δ, and selecting the Δ with optimal ordering closest to a ‘perfect’ ordering where all samples from a given species appear contiguously. This requires defining a distance between orderings. Let *X*_*i*_ be the set of samples of species *i* and *s* be the total number of species, *n*_*i*_ = |*X*_*i*_| be the number of samples of species *i*, and $y_{u}^{\alpha }$ be the position of sample *u* in an ordering *α*. Let the sum of pairwise absolute differences in sample positions of species *i* in ordering *α* be denoted by $f_{i}\left(\alpha \right)=\frac{1}{2}\sum_{\left(u,v\right)\in X_{i}{}^{2}}\left|y_{u}^{\alpha }-y_{v}^{\alpha }\right|$. It can be shown that this function is minimised if and only if *α* is a perfect ordering with respect to species *i*, with value $f_{i}\left(\alpha \right)=n_{i}\left(n_{i}^{2}-1\right)/6$. We define the distance between two orderings *α*, *β* as $d\left(\alpha ,\beta \right)=\sum_{i=1}^{s}\left|f_{i}\left(\alpha \right)-f_{i}\left(\beta \right)\right|$. It can be shown that *d* is a pseudometric on the space of sample orderings, satisfying symmetry and the triangle inequality. Then, the scoring function $g\left(\alpha \right)=\sum_{i=1}^{s}\left(f_{i}\left(\alpha \right)-n_{i}\left(n_{i}^{2}-1\right)/6\right)$ measures the distance of an ordering from a perfect ordering, with *g*(*α*) = 0 if and only if *α* is a perfect ordering, wherein samples of any given species are contiguously positioned. Meanwhile *g*(*α*) > 0 indicates that samples of some species are interleaved with samples from other species in the ordering *α*. Consequently, the optimal value of Δ is the one that induces an ordering *α* with the smallest *g*(*α*). Note that more than one Δ may induce the same smallest score, in which case we select the minimum Δ attaining this score. Figs 6 and 7 depict heatmaps with optimal values Δ = 7 and Δ = 8, respectively, optimised over Δ ∈ {1,2, …,100}. We remark that Δ = 8 induces a perfect ordering in Fig 7.

Agglomerative clustering was performed using *SciPy* [26], and heatmaps were generated using *seaborn* [27] and *Matplotlib* [28], in *Python*.

## Results

### The FcMBL method is highly sensitivity for a range of organisms causing BSIs

**Fig 4:**
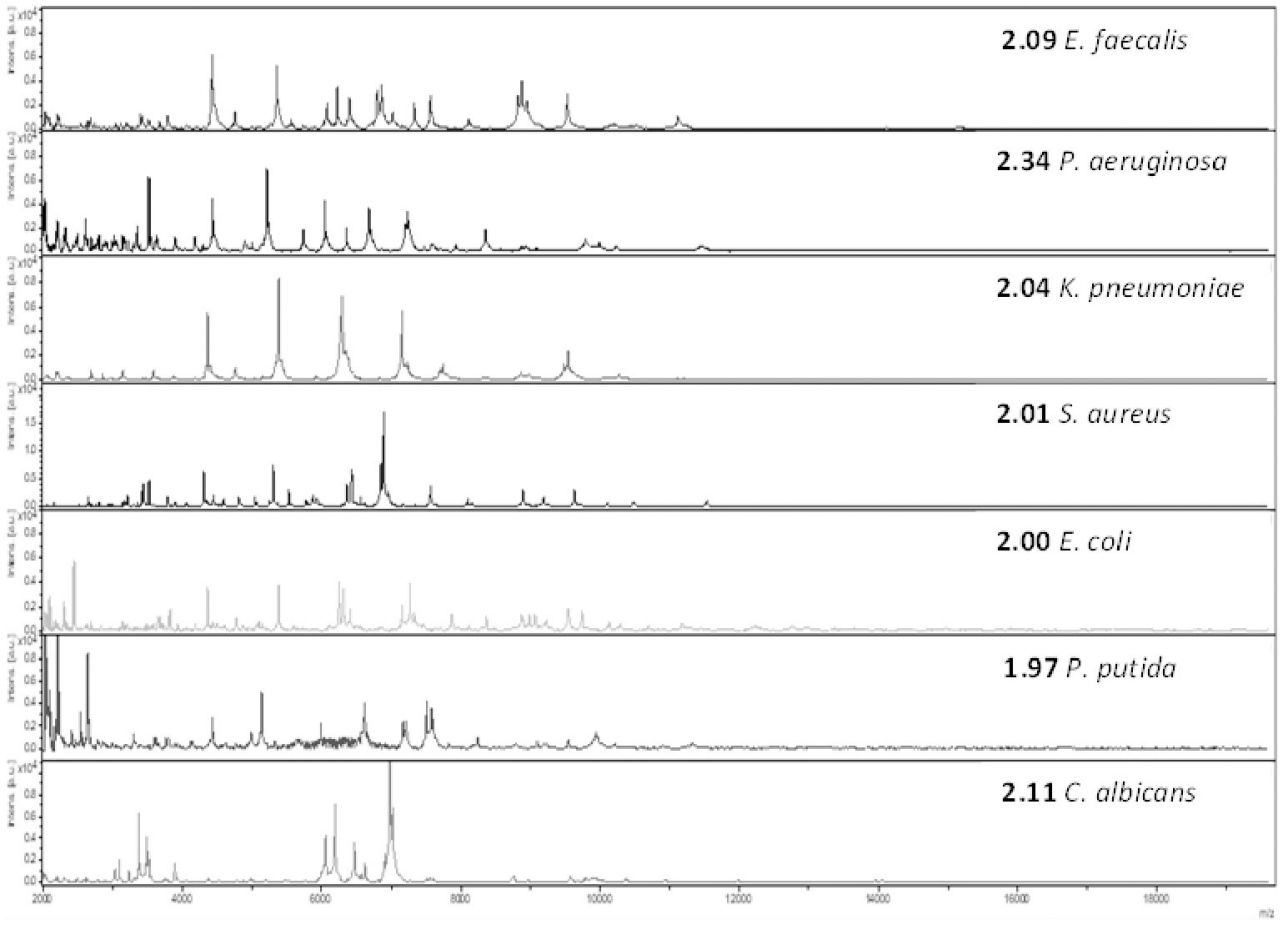
MALDI-TOF MS spectra of bacteria and yeast captured and eluted from FcMBL-processed beads. Bruker Biotyper log scores in bold.

The FcMBL method rapidly captured and detected a range of clinically relevant bacteria and yeast from spiked BCs and produced high Biotyper log scores (Fig 4). The FcMBL method showed high sensitivity in detecting clinical paediatric infections from positive BCs at GOSH with 64 of 68 (94.1%) samples being positively identified using MALDI-TOF MS (Table 1). These samples comprised 14 (30%) Gram-negative bacteria, 51 (63.9%) Gram-positive bacteria, and 3 (8%) yeast. Of the 68 tested by the FcMBL method, 64 (94.1%) were identified to the genus level (≥1.70) and 41 (60.3%) to the species level (≥2.00). For the FcMBL method, higher Biotyper log scores were generally seen with Gram-negative bacteria (average score 2.20) than Gram-positive bacteria (average score 1.91) and yeast (average score 2.00).

**Table 1:** MALDI-TOF MS identification of paediatric samples using FcMBL beads. Seven patients had both an anaerobic and aerobic bottle processed if available, accounting for the higher number of FcMBL positive samples seen for some species.

Species	No. of patient strains	No. of FcMBL positive	Bruker Biotyper Log score range
***Staphylococcaceae***			
*Staphylococcus aureus*	6	6	1.72–2.29
*Staphylococcus epidermidis*	9	8	0.00–1.92
*Staphylococcus haemolyticus*	2	2	1.77–1.82
*Staphylococcus hominis*	2	2	2.05
*Staphylococcus equorum*	1	1	1.70
*Staphylococcus capitis*	1	1	2.29
***Streptococcaceae***			
*Streptococcus oralis*	2	1	0.00–2.20
*Streptococcus mitis*	3	2	0.00–2.20
*Streptococcus parasanguinis*	2	3	1.87–2.34
*Streptococcus perosis*	1	1	2.00
*Streptococcus pneumoniae*	2	3	2.04–2.13
***Enterococcaceae***			
*Enterococcus faecium*	4	4	1.71–2.30
*Enterococcus faecalis*	3	4	2.00–2.11
***Enterobacteriaceae***			
*Escherichia coli*	2	1	0.00–1.95
*Enterobacter cloacae group*	2	3	1.73–2.09
*Raoultella ornithinolytica*	1	2	2.12–2.18
*Klebsiella oxytoca*	2	3	2.03–2.39
*Klebsiella pneumoniae*	3	3	2.21–2.31
**Other species**			
*Micrococcus luteus*	1	1	1.96
*Stenotrophomonas maltophilia*	3	3	2.15–2.28
*Burkholderia cepacia*	1	1	2.13
*Pseudomonas aeruginosa*	2	2	2.34–2.36
*Clostridium perfringens*	1	1	1.86
*Actinomyces graevenitzii*	1	1	1.88
**Yeast**			
*Candida glabrata*	1	1	2.00
*Candida albicans*	1	1	1.79
*Candida parapsilosis*	2	2	1.87–2.18
*Wickerhamomyces anomalus*	1	1	2.14
	**61**	64/68	**94.1%**

### Comparison of the Sepsityper^®^ and FcMBL protocols for clinical blood cultures

Of the 61 patients enrolled, 25 had a Sepsityper^®^ analysis performed as part of their routine diagnostic standard operating procedure (Fig 2). The Sepsityper^®^ identified only 17 (68%) to genus and 10 (40%) to species level confidence compared to the FcMBL method which was superior with 25 (100%) and 12 (48%) respectively (Table 2). A McNemar’s test indicated a statistically significant improved identification to genus level (*P* = 0.0078) with the FcMBL method, but not to species level (*P* = 0.5488). Wilcoxon’s signed-rank test indicated a statistically significant increase in log score with the FcMBL method (*P =* 0.0179).

**Table 2:** Bruker Biotyper log score comparison of the Sepsityper^*®*^ and FcMBL methods for the detection of pathogens in positive clinical blood culture samples. Bold Log scores indicate the higher score of the two methods.

	Standard Biotyper module	
Species	Sepsityper method	FcMBL method
*Staphylococcus aureus*	**2.29**	2.04
*Staphylococcus aureus*	2.10	**2.22**
*Staphylococcus aureus*	**2.18**	2.05
*Staphylococcus aureus*	**2.34**	2.14
**Coagulase-negative staphylococci**		
*Staphylococcus epidermidis*	**1.78**	1.73
*Staphylococcus epidermidis*	Failed	**1.74**
*Staphylococcus epidermidis*	Failed	**1.76**
*Staphylococcus epidermidis*	Failed	**1.87**
*Staphylococcus epidermidis*	Failed	**1.76**
*Staphylococcus epidermidis*	**2.23**	1.86
*Staphylococcus capitis*	1.72 (*Staphylococcus hominis*)	**2.29**
**Streptococcaceae**		
*Streptococcus oralis*	1.83 (*Streptococcus mitis*)	**2.20**
*Streptococcus mitis*	1.78 (*Streptococcus pneumoniae*)	**2.20**
*Streptococcus mitis*	**2.02**	1.72
*Streptococcus pneumoniae*	**2.35**	1.91
*Streptococcus pneumoniae*	Failed	**1.77**
*Streptococcus salivarius*	Failed	**2.09**
**Enterococcaceae**		
*Enterococcus faecium*	**2.11**	1.79
*Klebsiella oxytoca*	**2.18**	2.03
**Other species**		
*Micrococcus luteus*	1.90	**1.96**
*Stenotrophomonas maltophilia*	Failed	**2.25**
*Stenotrophomonas maltophilia*	2.12	**2.15**
*Burkholderia cepacia*	1.79	**2.13**
**Yeast**		
*Wickerhamocytes anomalus*	Failed	**2.14**
*Candida albicans*	Failed	**1.79**
*Candida parapsilosis*	1.85	**1.87**
**Identification to genus**	**17/26 (68%)**	**26/26 (100%)**
**Identification to species**	**10/26 (38%)**	**13/26 (50%)**

**Fig 5:**
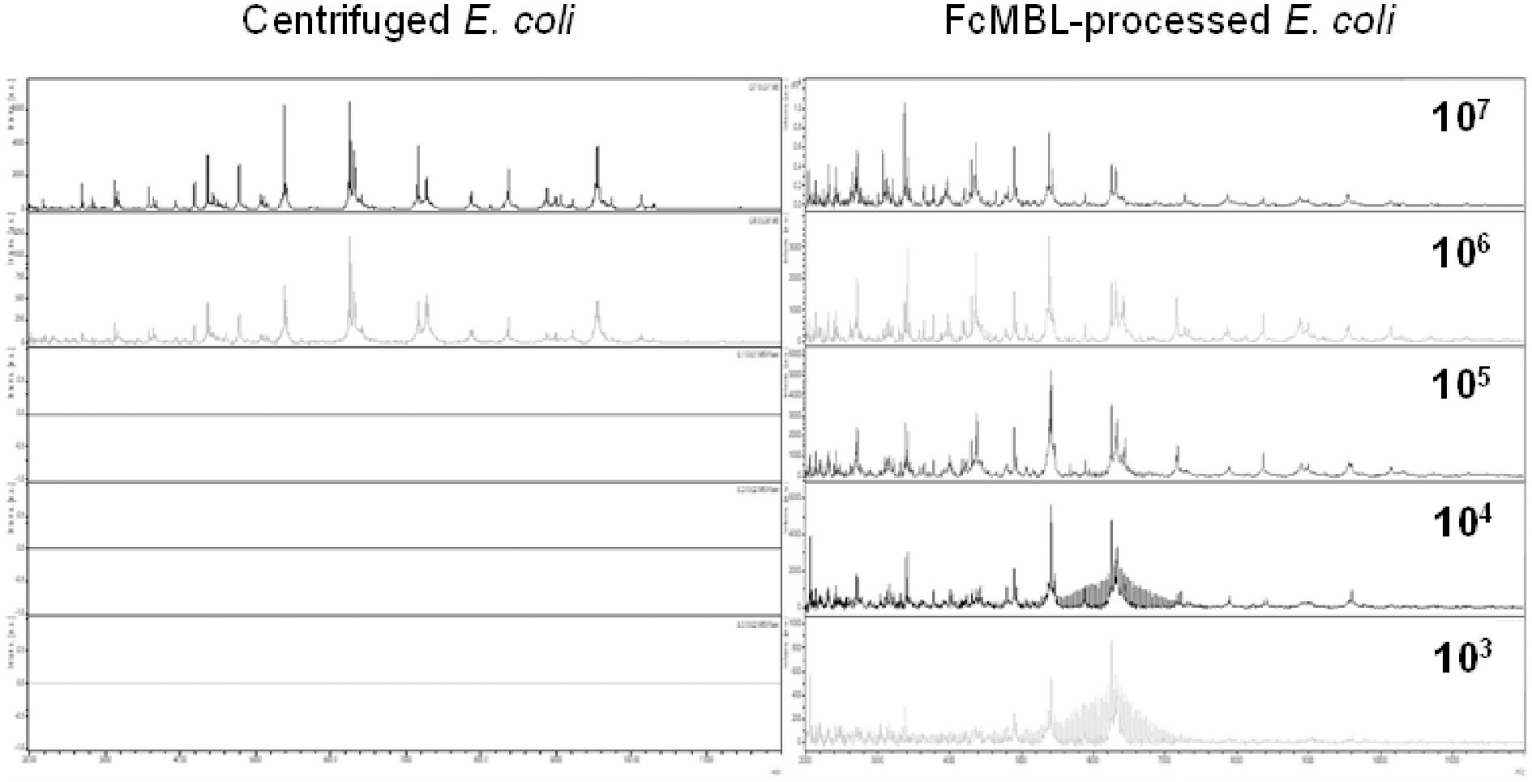
Extreme enrichment of *E*. *coli* from spiked blood samples compared to centrifuged samples. *E*. *coli* specific peaks are found in all concentrations using the FcMBL method. The limit of detection for the centrifuged samples was found to be at 10^6^ CFU/ml.

To investigate the advantage of magnetic bead enrichment over centrifugation methods, we then carried out further comparisons between centrifugation techniques versus the FcMBL method (Fig 5). Using spiked samples, we found that the centrifugation method alone was unable to detect low concentrations of bacteria, and bacterial peaks below concentrations of 10^6^/mL were only observed using FcMBL enrichment, which enabled detection of *E*. *coli* specific peaks at concentrations as low as 10^3^/mL.

### Comparison of the Sepsityper^®^ versus the FcMBL protocol for the detection of candida species

Additionally, due to the low incidence of candidemia (5) seen in the clinic during this time, spiked candida samples were prepared and processed with both the Sepsityper^®^ and FcMBL methods (Table 3). The Sepsityper^®^ method detected 17 (70.8%) of the 24 samples, with 6 (25%) identified to species level. The FcMBL method showed superior performance and was able to detect 24 (100%) of the candida samples to genus level, with 9 (37.5%) species level identification. A McNemar’s test indicated a statistically significant improved identification to genus level (*P* = 0.0156) with the FcMBL method, but not species level (*P* = 0.5811). Wilcoxon’s signed-rank test indicated a statistically significant increase in log score with the FcMBL method (*P =* 0.0153).

**Table 3:** Comparison of Bruker Biotyper log scores between the Sepsityper^®^ and FcMBL rapid identification methods for fungemia. Log scores highlighted in red indicate scores below identification confidence. Bold log scores indicate the higher score of the two methods.

	Standard Biotyper module	
Organism	Sepsityper Log Score	FcMBL Log Score
*C*. *albicans*		
1	1.66	**1.81**
2	0.00	**1.86**
3	1.83	**1.97**
4	**2.06**	1.83
5	1.36	1.74
6	1.87	**1.90**
*C*. *kefyr*		
1	1.98	**2.31**
2	**2.17**	2.02
3	1.98	**2.24**
*C*. *krusei*		
1	1.93	**2.05**
*C*. *auris*		
1	0.00	**1.87**
2	1.13	**1.92**
3	1.89	**2.19**
*C*. *glabrata*		
1	0.00	**1.93**
2	1.97	**2.26**
3	**2.20**	1.92
*C*. *tropicalis*		
1	**2.02**	1.93
*C*. *parapsilosis*		
1	0.00	**1.72**
2	1.96	**2.07**
3	1.96	**2.22**
4	**2.07**	1.77
*C*. *guilliermondii*		
1	1.94	**2.01**
2	1.79	**1.92**
3	**2.02**	1.72
**Genus level**	**17/24 (70.8%)**	**24/24 (100%)**
**Species level**	**6/24 (25%)**	**9/24 (37.5%)**

**Fig 6:**
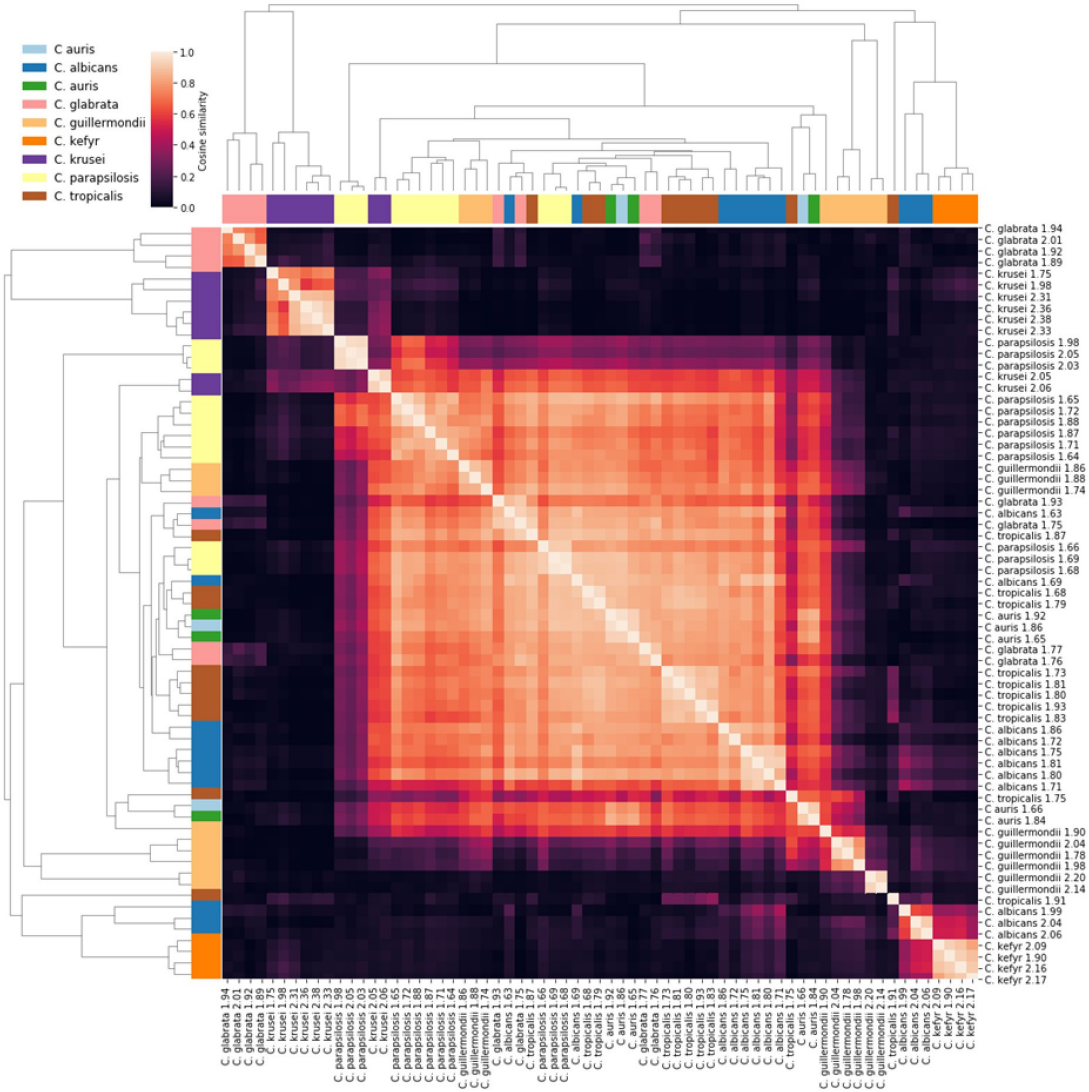
Heatmap of spectral similarities with dendrogram clustering analysis for FcMBL candida mass spectra (2,000–20,000m/z).

**Fig 7:**
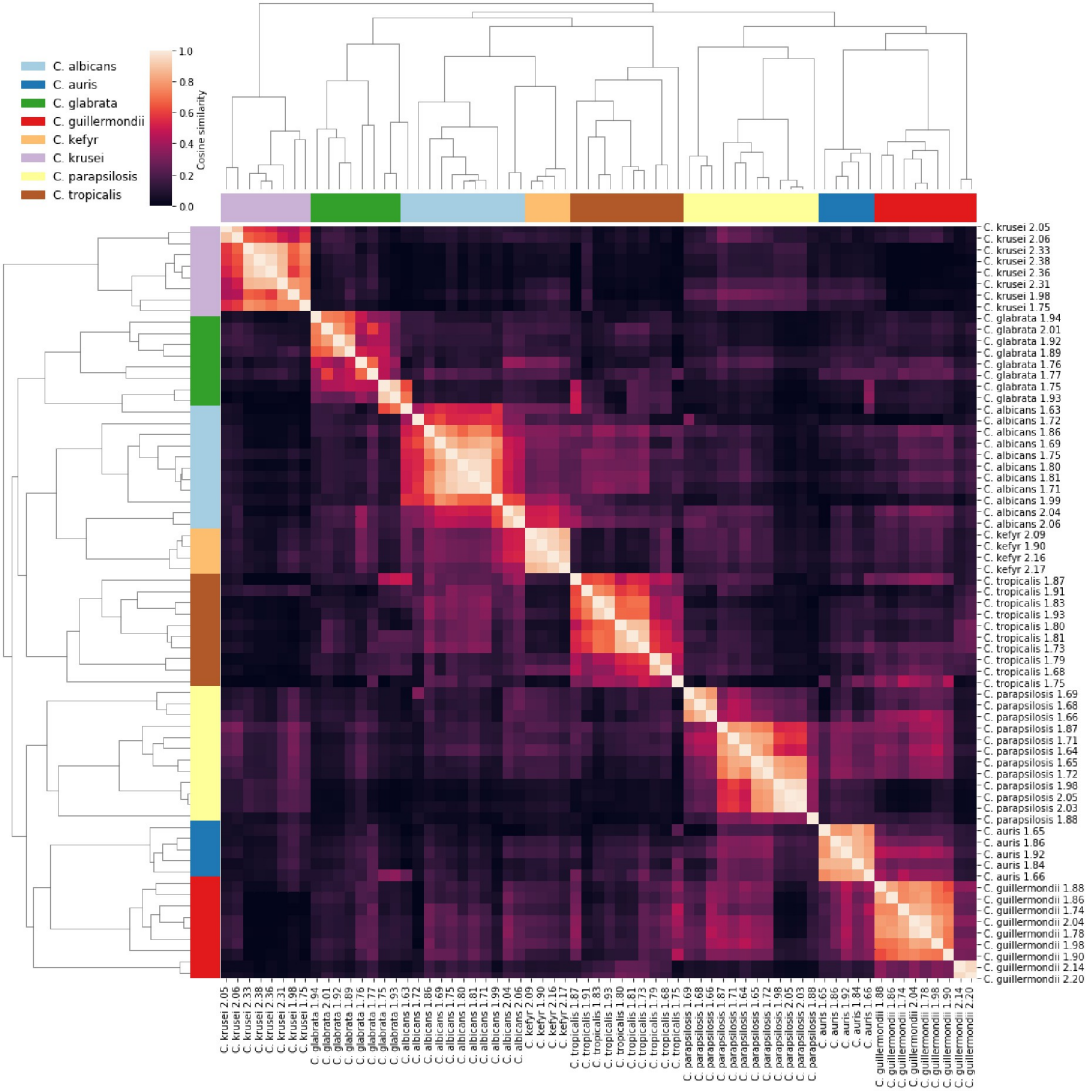
Heatmap of spectral similarities with dendrogram clustering analysis for FcMBL candida mass spectra (2,500–7,400m/z).

As relatively low species level identification was observed with candida compared to bacteraemia, the FcMBL spectra from spiked candida samples were collected and analysed. The mass spectra at full (2,000–20,000*m/z*) and truncated (2,500–7,400*m/z*) ranges were compared using a clustering analysis (Figs 6 and 7). Increased clustering of species was seen using the truncated *m/z* range where several apparent FcMBL-specific background peaks are removed, suggesting that an FcMBL-specific library may achieve greater species level discrimination.

## Discussion

The engineered FcMBL protein has demonstrated a range of successes with regards to pathogen capture, from filtering septic blood via haemodialysis [22, 29] to the detection of systemic infections by quantifying pathogen-associated molecular patterns (PAMPs) in the blood of septic patients [19, 20]. In this study, our novel in-house method using FcMBL to diagnose BSIs direct from clinical BCs demonstrated a higher sensitivity than the standard Sepsityper^®^ diagnostic kit. By capitalising on the FcMBL-coated beads’ comprehensive recognition of bacteria and yeast, this study highlights a new method with high sensitivity, which reduces turnaround time and enhances sensitivity of pathogen diagnostics needed for a paediatric hospital regularly managing children with a wide range of unusual infections. Both the FcMBL and Sepsityper^®^ methods can be performed in under 30 minutes using materials and reagents familiar to clinical microbiology laboratories. The FcMBL method also uses both the Bruker Biotyper, a commercially available platform for clinical microbiology laboratories across the UK, and the same extraction techniques as the Sepsityper^®^. Thus, the FcMBL method introduced here would be readily adoptable in existing clinical settings.

We found the FcMBL method to have higher sensitivity identifying Gram-positive bacteria and yeast using MALDI-TOF MS than the Sepsityper^®^. Though less clinically common than bacteraemias, candidemia carries a disproportionately high mortality rate, further highlighting the need for rapid identification [30]. Various studies demonstrate a limited ability (~57–82% success) to detect yeast using the Sepsityper^®^ method [31, 32]. To remedy this, we used FcMBL-conjugated beads to bind and enrich both whole microbes and microbial debris from BC samples for improved diagnostic sensitivity. Sepsityper^®^ results from the clinic were compared to the FcMBL identification assessment (Table 2). FcMBL captured and concentrated microbes for MALDI-TOF MS analysis more effectively than the Sepsityper^®^. The Sepsityper^®^ detected only 68% of samples to genus level and 40% to species level compared to 100% and 48% by the FcMBL method. Of all 25 Sepsityper^®^-confirmed BC samples, FcMBL detected all Gram-negative bacteria (4), Gram-positive bacteria (18), and yeast (3). In contrast, the Sepsityper^®^ failed to detect 2/3 of the yeast samples as well as 5/18 Gram-positive and 1/4 Gram-negative bacterial samples.

Compared to centrifugation methods described, the FcMBL method uses magnetic beads to enable extreme enrichment of microbes and their fragments to detect even low concentrations of bacterial proteins (Fig 5). The average CFU of positive BCs for yeast species has been shown to be approximately 5.9x10^5^, compared to 3.2x10^8^ and 2.6x10^8^ for Gram-negative and Gram-positive bacteria, respectively [33, 34]. MALDI-TOF MS benchtop systems such as the Bruker Biotyper have demonstrated a detection range of >10^5^–10^6^, though some studies demonstrate as low as >10^4^ [35, 36]. This lower yield of yeast in cultures may explain the inconsistent results with the Sepsityper^®^ method. By capturing whole cell and fragments and enriching concentrations, FcMBL beads improve the sensitivity for detecting lower yielding organisms such as yeast.

Due to the generally low prevalence of fungemia in the general population, we performed an inoculation study to further explore whether FcMBL enrichment improved fungal identification, as compared to the Sepsityper^®^. Volunteer BCs were spiked with previous candidemia isolates from GOSH. We found the FcMBL method to be highly sensitive for identifying candidemia by MALDI-TOF MS (Table 3). The FcMBL method identified 100% of samples to genus level and 37.5% to species level, in contrast to only 70.8% and 25%, respectively, for the Sepsityper^®^. Although the FcMBL method did not identify all the samples to species level, the Biotyper provided the correct species identification. We directly inspected the mass spectra to identify potential background peaks from the FcMBL complex. A study in 2021 demonstrated that background peaks from the FcMBL molecule can affect the Biotyper log scores produced by the Bruker Biotyper algorithm [18]. Identification scores could therefore be improved by modifying libraries and algorithms to account for the background introduced by FcMBL enrichment. With this insight, we performed a cluster analysis on the original full-length spectra used by the Biotyper and a truncated spectra with several apparent FcMBL-specific background peaks removed (Figs 6 and 7). We confirmed that candida species clustered significantly more effectively when truncated, highlighting the potential for a further increase in performance with FcMBL-tailored libraries.

In summary, we have introduced a new method for rapid BSI diagnosis using FcMBL beads and demonstrated that this method can capture a wide range of clinical pathogens, allowing for rapid identification by MALDI-TOF MS without the need for subculture. Despite its lower incidence, candidemia is associated with particularly high mortality, and the slow-growing nature of yeast in culture limits the ability of existing diagnostic methods to yield timely pathogen identification. Having a highly efficient and rapid method to detect yeast, especially in paediatric settings, is essential for optimal clinical management. With increasing rates of antifungal resistance, appropriate clinical management changes are required to prevent mortality cases rising and ease economic burden. With this, the ability of FcMBL beads to bind a wide range of clinically relevant whole cell microbes and PAMPs in blood promises to revolutionise clinical microbiology and infection control.
